# Expression of phosphate and calcium transporters and their regulators in parotid glands of mice

**DOI:** 10.1007/s00424-022-02764-x

**Published:** 2022-10-24

**Authors:** Seraina O. Moser, Betül Haykir, Catharina J. Küng, Carla Bettoni, Nati Hernando, Carsten A. Wagner

**Affiliations:** Institute of Physiology, University of Zürich, 8057 Zurich, Switzerland

**Keywords:** Phosphate, Salivary glands, Slc34, Slc20, PTH, FGF23

## Abstract

The concentration of inorganic phosphate (Pi) in plasma is under hormonal control, with deviations from normal values promptly corrected to avoid hyper- or hypophosphatemia. Major regulators include parathyroid hormone (PTH), fibroblast growth factor 23 (FGF-23), and active vitamin D_3_ (calcitriol). This control is achieved by mechanisms largely dependent on regulating intestinal absorption and renal excretion, whose combined actions stabilise plasma Pi levels at around 1–2 mM. Instead, Pi concentrations up to 13 and 40 mM have been measured in saliva from humans and ruminants, respectively, suggesting that salivary glands have the capacity to concentrate Pi. Here we analysed the transcriptome of parotid glands, ileum, and kidneys of mice, to investigate their potential differences regarding the expression of genes responsible for epithelial transport of Pi as well as their known regulators. Given that Pi and Ca^2+^ homeostasis are tightly connected, the expression of genes involved in Ca^2+^ homeostasis was also included. In addition, we studied the effect of vitamin D_3_ treatment on the expression of Pi and Ca^2+^ regulating genes in the three major salivary glands. We found that parotid glands are equipped preferentially with *Slc20* rather than with *Slc34* Na^+^/Pi cotransporters, are suited to transport Ca^2+^ through the transcellular and paracellular route and are potential targets for PTH and vitamin D_3_ regulation.

**Supplementary Information:**

The online version contains supplementary material available at 10.1007/s00424-022-02764-x.

## Introduction

Salivation plays several roles in the gastrointestinal tract, including hydration of epithelia, initiation of digestion, and defence against microbes (for review, see [51]). Most of the daily salivary output is produced in response to eating, whereas salivation rate is slow between meals and virtually absent during sleep. Mammals have three major pairs of salivary glands, namely submandibular, sublingual and parotid. As other secretory tissues, salivary glands contain two types of epithelial cells: acinar cells that are responsible for secretion of the primary saliva, and ductal cells accountable for the secretion/reabsorption processes that determine the composition of the final saliva. In acinar cells, transepithelial Cl^−^ secretion together with paracellular Na^+^ transport generates the osmotic gradient required for water secretion into the acinar lumen. Acinar Cl^−^ secretion is supported by basolateral uptake of Cl^−^, mediated by Na^+^/K^+^/Cl^−^ cotransporters and Cl^−^/HCO_3_^−^ exchangers, followed by apical efflux via a Ca^2+^-dependent Cl^−^-channel [17, 44, 57], whereas Na^+^ and water transport are mediated by claudin-2 and aquaporin-5, respectively [35]. As this plasma-like isotonic primary secretion travels along the duct, ductal cells reabsorb most of the NaCl by mechanisms involving the apical Na^+^ and Cl^−^ channels ENaC and CFTR, respectively, as well as a Cl^−^/HCO_3_^−^ exchanger, whereas they secrete K^+^ probably via apical Slo K^+^ channels [48, 55, 62, 64]. Since ductal cells are rather impermeable to water, these changes in electrolyte composition result in an alkaline hypotonic final saliva (for review, see [10, 51]).

The reported concentration of Pi in saliva ranges from 1.3 to 13 mM in humans [60] whereas up to 40 mM have been reported in ruminants [38], suggesting that salivary glands have the capacity to concentrate Pi. Furthermore, salivary Pi was recently reported to be increased in patients undergoing hemodialysis [61] and to correlate with carotid intima media thickness, a marker of atherosclerosis [47]. The mechanisms responsible for Pi transport in salivary glands have been studied mostly in ruminants. Unlike in other mammals, handling of Pi in ruminants involves a so-called endogenous Pi recycling, consisting of a large delivery of Pi into the gastrointestinal tract via salivary secretion followed by downstream Pi absorption mostly in the jejunum (for review, see [54]). This recycling mechanism provides high amounts of Pi to the forestomach which are required to support microbial growth as well as to increase the intestinal buffering capacity, since ruminal microbial fermentation produces large quantities of short-chain fatty acids. In ovine, the acinar cells of the parotid glands are known to secrete large amounts of Pi, a process that is initiated by uptake of Pi across the basolateral membrane by a Na^+^-dependent and phosphonoformic acid-inhibitable mechanism attributed to NaPi-IIb/*Slc34a2* [38, 66, 72], followed by apical extrusion mediated by a mechanism which molecular identity remains unknown (for review, see [54]). Transport of Pi across basolateral membrane vesicles isolated from ovine parotid glands is not regulated by dietary Pi [37, 66]. This is unlike transport of Pi into intestinal brush border membranes vesicles (BBMV), a process also mediated by NaPi-IIb whose intestinal expression is regulated by the dietary content of Pi [27, 30].

Expression of NaPi-IIb was confirmed in adult human parotid and submandibular glands obtained from resection material of patients undergoing maxillofacial surgery [34]. In both glands, NaPi-IIb was detected in acinar and ductal cells, with the expression in acinar cells restricted to the basolateral membrane whereas apical expression was found along ducts. More recently, the expression of NaPi-IIb at the apical membrane of submandibular ductal cells was also described in mice [40]. However, unlike previous reports in ruminants, the salivary Pi concentration in mice reflects the dietary content of Pi, with higher salivary Pi levels in mice fed for a week with high Pi diets than those fed on low Pi [40]. Moreover, a high Pi diet (as well as an intragastric Pi bolus) reduces the expression of NaPi-IIb at the apical membrane of sublingual and/or submandibular ductal cells in mice. However, no NaPi-IIb/*Slc34a2* transcripts were detected in a recently published single-cell RNA-seq analysis in which different cell populations of mice submandibular glands were investigated [67]. Unlike Pi, the salivary concentration of Ca^2+^ is slightly lower than its plasma concentration [60].

Here, we performed RNA-seq to compare the expression of Pi transporters and regulatory genes in mouse parotid glands with their expression in ileum and kidney, the two epithelial tissues in which Pi transport is better studied. Because many of the regulators of Pi homeostasis also affect intestinal and renal Ca^2+^ handling, the expression of Ca^2+^ transporters was also investigated. In addition, the effect of 3 days vitamin D_3_ treatment was examined since this treatment is known to regulate Pi and Ca^2+^ transport in kidney and intestine.

## Material and methods

### Animal handling and sample collection

Wild type male mice (C57BL/6J) 10–12 weeks old were purchased from Janvier (Genest Saint Isle; France) and let to adapt for 1 week, during which they were fed ad libitum with standard chow (maintenance rodent diet 3436, Promivi Kliba AG with 0.8% phosphate and 1% calcium, 1000 IU vitamin D_3_) and held in individually ventilated cages. Then, tissue samples of 5 mice were extracted and used for RNA quantification whereas 5 mice were fixed with paraformaldehyde (PFA) for immunofluorescence studies. Additionally, 12 mice were injected intraperitoneally during two consecutive days (once per day, in the morning) with either vehicle (corn oil/ethanol/PBS) or 4 μg kg^−1^ BW of 1,25(OH)_2_ vitamin D_3_ (6 animals per group) and tissue samples were extracted for RNA quantification.

Mice were anaesthetized with isoflurane (Piramal Critical Care Deutschland GmbH. Hallbergmoos, Germany) and killed by cervical dislocation prior to collection of kidneys, ileum, and salivary glands (submandibular, parotid and sublingual). Kidneys were decapsulated whereas the ileum was rinsed with 0.9% NaCl and inverted in order to scrape off the epithelial layer. All samples were snap-frozen in liquid nitrogen and stored at – 80 °C. For fixation, mice were perfused through the left ventricle with 15 ml of a solution containing 5000 U/ml heparin, 1% lidocaine, 16% CaCl_2_, and 0.9% NaCl in distilled H_2_O, followed by 40 ml of 4% PFA in PBS. Tissues were extracted and kept overnight at 4 °C in 4% PFA and then sequentially incubated in 10%, 20%, and 30% sucrose/PBS during the following 2 days. Upon embedding in OCT media (Sakura) samples were frozen in liquid propane and stored at − 80 °C. Animal experiments were performed according to the Swiss law of animal welfare and were approved by the local veterinary authorities (Kantonales Veterinäramt Zürich) under the license number ZH240/19.

### RNA extraction, reverse transcription, and real-time quantitative PCR (qPCR)

One of each of the sublingual, submandibular, and parotid glands as well as half a kidney and a piece of the ileum epithelial scrapping were lysed in RT buffer containing 1% β-mercaptoethanol, using MagNA Lyser Green Beads and the Precellys 24 tissue homogeniser (Bertin Corp., Rockville, MD, USA) at 5500 rpm 2 × 20 s. RNA was purified with NucleoSpin RNA Column (Macherey–Nagel) and eluted in RNase free H_2_0. Aliquots of 300 ng were reverse transcribed into cDNA using the TaqMan Reverse Transcription kit (ThermoScientific, USA). The incubation protocol consisted of 10 min at 25 °C, 30 min at 48 °C and 5 min at 95 °C. The expression of the genes of interest was analysed by qPCR using the KAPA Probe Fast qPCR Universal Master Mix (2 ×) Kit (Kapa Biosystems. Cape Town, South Africa) in the presence of gene-specific FAM/TAMRA-labelled probe (0.1 µM) and primers (1 µM) (Supplementary Table 1). In few cases, SYBR green, instead of labelled probes, was used for quantification. The qPCR protocol consisted of 20 s incubation at 95 °C followed by 40 cycles of 3 s at 95 °C and 30 s at 60 °C, using the 7500 Fast Real-Time PCR System (Applied Biosystems, Switzerland). Cycle threshold (Ct) were manually set at the exponential phase of the amplification curve. Ct values of the analysed genes were normalised to that of the hypoxanthine–guanine phosphoribosyltransferase (*Hprt*) gene, and relative mRNA expression levels were calculated as 2^(Ct_*Hprt* –Ct_*gene*)^. Primers/probes were designed with the Primer BLAST tool of the National Center of Biotechnical Information (NCBI) and ordered from Microsynth (Balgach, Switzerland).

### RNA-seq

Transcriptome sequence analysis of RNA samples from parotid glands, kidney and ileum was done at the Functional Genomics Centre Zürich (FGCZ). Libraries were prepared using the Illumina TruSeq mRNA method and sequenced with the Illumina NovaSeq6000 system. Original reads were cleaned by removing adapter sequences, trimming low quality ends, and filtering reads with low quality (phred quality < 20) using Fastp (Version 0.20) [11]. Then, cleaned reads were aligned to the Mouse reference genome (build GRCm38.p5) and mRNA expression was quantified using Kallisto (Version 0.46.1) [9].

### Immunohistochemistry

Cryopreserved salivary glands and ileum samples were cut into 2–3 μm thin slices at – 25 °C using a Cryostat CM1850 (Leica) and mounted on pre-cooled SuperFrost Plus Slides (Mezel GmbH). Samples were rehydrated in PBS and incubated with 50 mM NH_4_Cl/PBS for 20 min at room temperature. For antigen retrieval, sections were then treated with either Tris–EDTA, pH 9 (10 mM Tris base, 1 mM EDTA, 0.05% Tween20) for 10 min at 95 °C or with 1% SDS/PBS for 5 min at room temperature. Bovine serum albumin (BSA) at 1% in PBS was used for blocking as well as for dilution of antibodies. Sections were incubated at 4 °C overnight with 1: 100 dilutions of a commercial (NPT2b11-A, Alpha Diagnostic International, San Antonio, TX, USA) or a homemade antibody against NaPi-IIb [30]. After washing the slides sequentially with hypertonic and isotonic PBS, samples were incubated for 1 h at room temperature in the dark with secondary anti-rabbit antibodies (1:1000, Alexa Fluor 488, A21202, Molecular Probes). All samples were co-stained with DAPI (1:500, D3571, Invitrogen). After washing, sections were embedded with DAKO-Glycergel mounting media (DAKO) and stored at 4 °C. Images were taken with a fluorescence microscope (Leica) and processed in ImageJ.

### Statistical analysis

Statistical analysis of gene expression in salivary glands from mice injected with vehicle versus vitamin D_3_-treated mice was done with GraphPad Prism8 (GraphPad Software, San Diego, USA). For each gland, differences between both groups were analysed with the unpaired Student’s *t* test. *p* ≤ 0.05 was considered significant. All data are presented as individual values with mean ± SD.

## Results

### Comparative mRNA expression of Na^+^/Pi cotransporters in salivary glands, ileum, and kidney

**Fig. 1:**
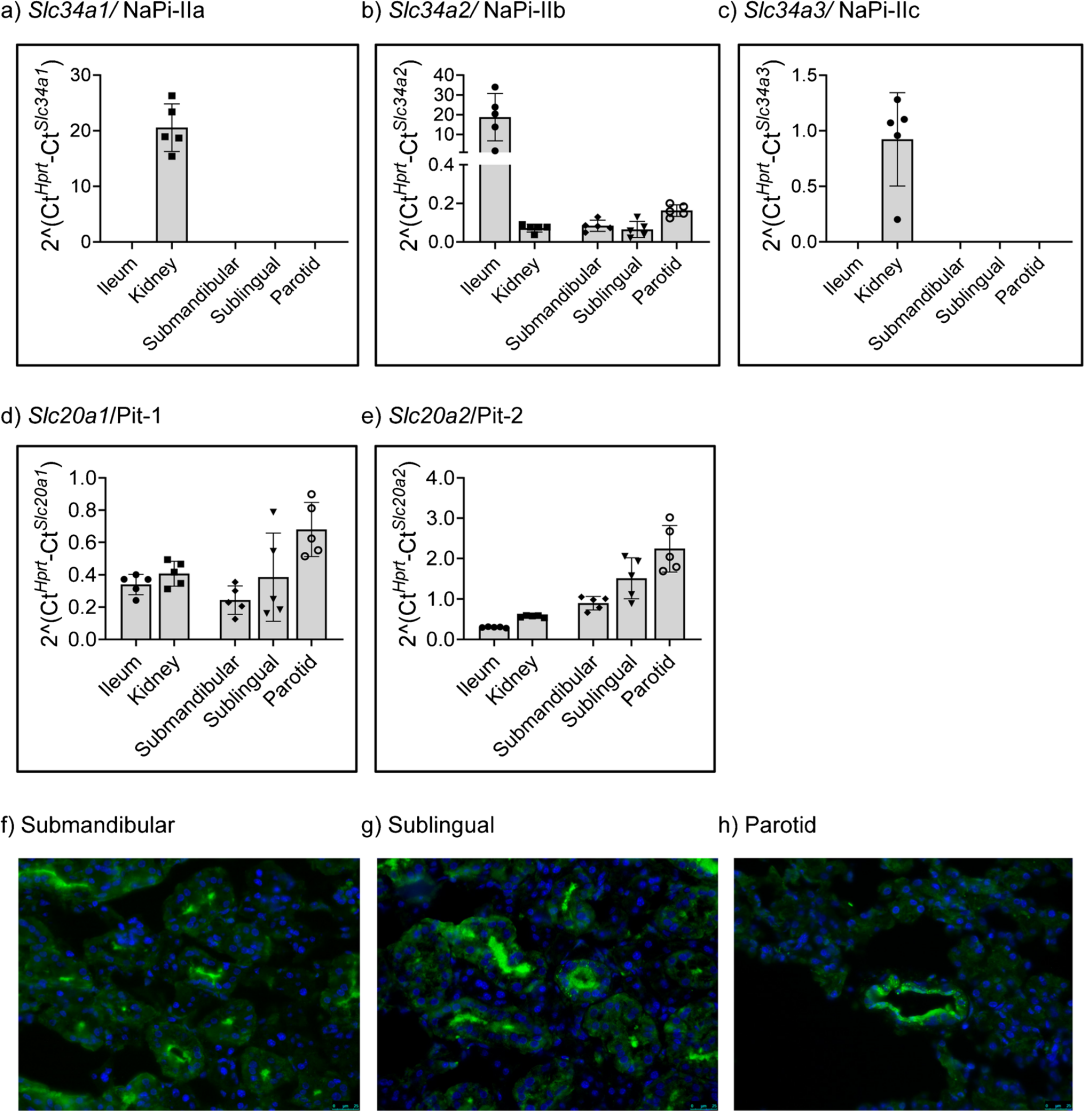
Expression of Na/Pi cotransporters mRNAs in salivary glands, ileum, and kidney. The mRNA expression of **a** *Slc34a1*, **b** *Slc34a2*, **c** *Slc34a3*, **d** *Slc20a1*, and **e** *Slc20a2* was quantified by qPCR in samples of ileum, kidney as well as from submandibular, sublingual, and parotid glands of mice (*n* = 5). Data was normalised to the expression of *Hprt*. Protein expression of NaPi-IIb/*Slc34a2* was analysed by immunofluorescence in sections of **f** submandibular, **g** sublingual, and **h** parotid glands. Scale = 25 µm

The mRNA abundance of *Slc34* and *Slc20* paralogues, the Na^+^/Pi cotransporters identified at the BBM of intestinal and renal epithelial cells (for review, see [18, 49]) was compared among the 3 types of salivary glands, ileum and kidney. Semi-quantitative measurements were performed by qPCR using primers with similar amplification efficiencies [53]. As previously reported [53], *Slc34a1/*NaPi-IIa and *Slc34a3/*NaPi-IIc mRNAs were detected in renal samples but not in ileum, and within kidney the abundance of *Slc34a1* was about 1 order of magnitude higher than *Slc34a3* (Fig. 1a, c); none of the 2 transcripts was detected in the different salivary glands. Also as anticipated, *Slc34a2/*NaPi-IIb mRNA was highly expressed in ileum, with 2.5 orders of magnitude lower levels also detected in kidney as recently reported [53] (Fig. 1b); low *Slc34a2* expression was observed in all salivary glands as well. As expected, *Slc20a1/*Pit-1 mRNA was expressed both in ileum and kidney (Fig. 1d), though at far lower levels than *Slc34a1* and *Slc34a2*; *Slc20a1* transcripts were also detected in salivary glands. The expression of *Slc20a2/*Pit-2 mRNA was also comparable in ileum and kidney (Fig. 1e), but in both tissues was well below the *Slc34a1 and Slc34a2* paralogues levels; *Slc20a2* transcripts were detected in the 3 salivary glands as well.

The expression of NaPi-IIb at the protein level was analysed in salivary glands by staining tissue sections with a home-made antibody that detects the cotransporter in mouse intestinal epithelia [30] (Supplementary Fig. 1). However, this antibody failed to provide reliable signals in any of the salivary glands (data not shown). Instead, a commercial antibody previously reported to stain NaPi-IIb in submandibular glands of mice [40] labelled ductal structures in all 3 glands (Fig. 1f-h), with the signal concentrated on the luminal side. We did not observe any basolateral staining. The specificity of this antibody was confirmed by performing immunofluorescence of intestinal samples, since it reacted with a luminal protein in sections of ileum of wild type but not of intestinal-specific NaPi-IIb-deficient mice (Supplementary Fig. 1).

### Transcriptome analysis of Na^+^/Pi cotransporters in parotid glands, ileum, and kidney

**Fig. 2:**
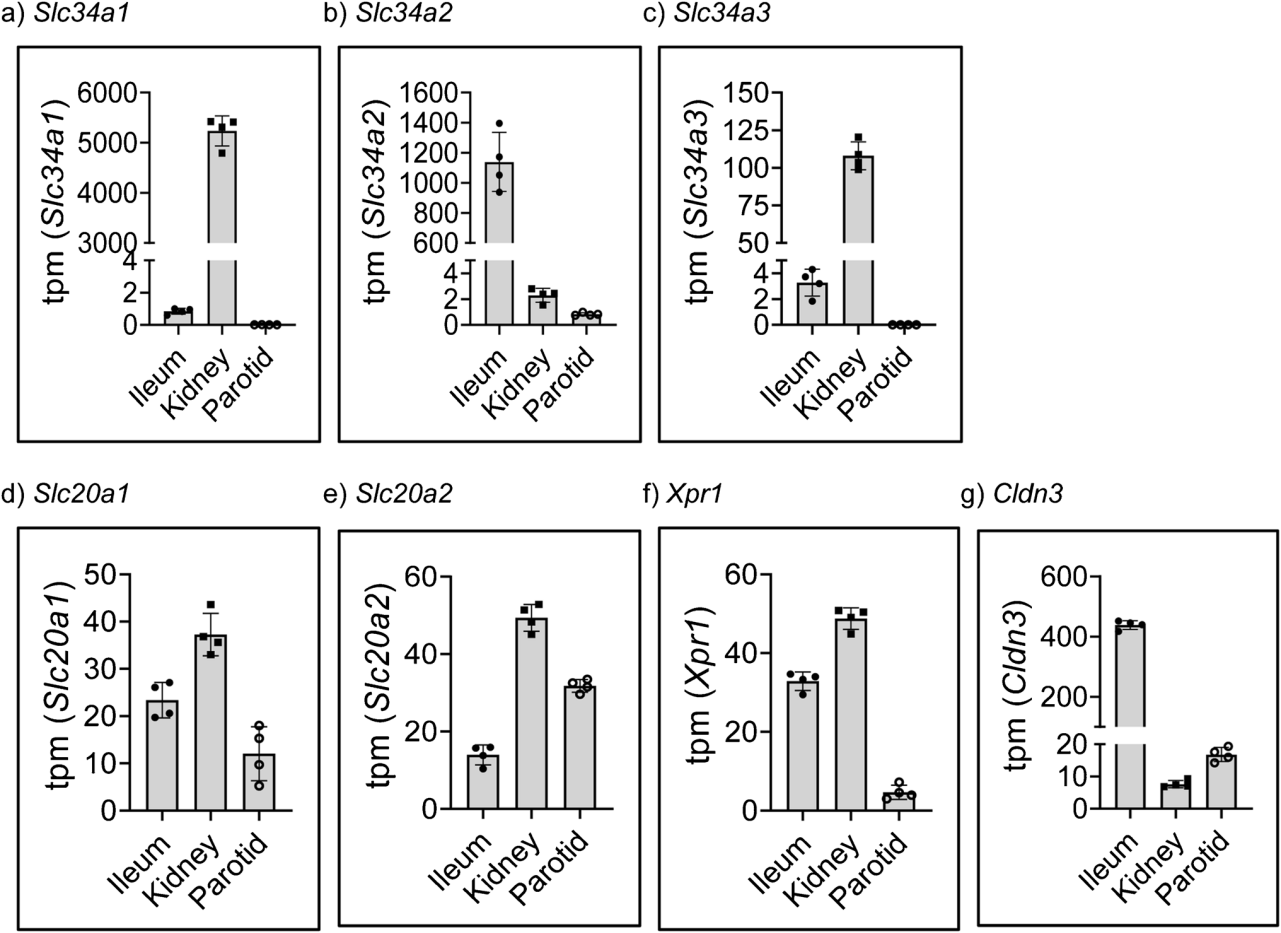
Transcriptome quantification of Na/Pi cotransporters, *Xpr1* and *Cldn3* in parotid glands, ileum, and kidney. Abundance of **a** *Slc34a1*, **b** *Slc34a2*, **c** *Slc34a3*, **d** *Slc20a1*, **e** *Slc20a2* as well as **f** *Xpr1*, the putative basolateral transporter and **g** *Cldn3*, a claudin suggested to tighten the intestinal epithelia for paracellular transport of Pi, in the RNAseq-based transcriptome analysis of ileum, kidney, and parotid glands (*n* = 4). Data is expressed as transcripts per kilobase million (tpm)

RNA-seq was performed in samples of parotid glands, ileum and kidney (Supplementary Table 2). From all salivary glands, parotids were chosen as they tended to have higher expression of Na^+^/Pi transporters than the other 2 glands (Fig. 1b, d and e). RNA-seq detected the presence of 15,210 transcripts in ileum, 16,008 in kidney and 15,609 in parotid glands (Supplementary Table 2). This analysis confirmed the relative mRNA expression of *Slc34* transporters in ileum and kidney provided by qPCR, i.e. the abundance of *Slc34a1/*NaPi-IIa (Fig. 2a) and *Slc34a3/*NaPi-IIc (Fig. 2c) transcripts was about 4 and 1.5 orders of magnitude higher in kidney than in ileum, respectively, whereas *Slc34a2/*NaPi-IIb mRNA expression was 2.5 orders of magnitude higher in intestine than in kidney (Fig. 2b). This analysis also confirmed the near absence of *Slc34* paralogues in parotid glands except for some low expression of *Slc34a2*, as expected based on the qPCR and immunofluorescence data described above (Fig. 1b, f-h). In contrast to *Slc34* paralogues, both *Slc20* transcripts were expressed in all analysed tissues, in agreement with their ubiquitous distribution, and differences between organs never reached 1 order of magnitude (Fig. 2d, e). *Xpr1*, the gene encoding a putative basolateral transporter for Pi [23], was expressed at comparably high levels in ileum and kidney, whereas its abundance was about 1 order of magnitude lower in parotid glands (Fig. 2f). The expression of *Cldn3*, a component of tight junctions suggested to seal the paracellular pathway for Pi in the gut [26], was almost 2 orders of magnitude higher in intestine than in kidney, with only low levels detected in parotid glands (Fig. 2g).

### Transcriptome analysis of genes involved in transport of Ca^2+^ in parotid glands, ileum, and kidney

**Fig. 3:**
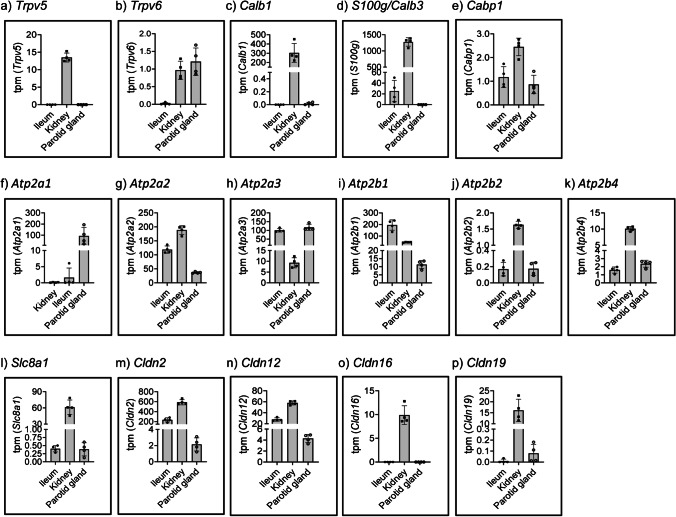
Transcriptome quantification of genes involved in Ca^2+^ transport in parotid glands, ileum, and kidney. Abundance of apical channels **a** *Trpv5* and **b** *Trpv6*, of intracellular Ca^2+^-binding proteins, **c** *Calb1*, **d** *S100g*, and **e** *Cabp1*, of sarcoplasmic reticulum ATPases **f** *Atp2a1*, **g** *Atp2a2*, and **h** *Atp2a3*, of basolateral Ca^2+^ ATPases **i** *Atp2b1*, **j** *Atp2b2*, and **k** *Atp2b4*, of basolateral Na^+^/Ca.^2+^ exchangers, **l** *Slc8a1*, and of claudins, **m** *Cldn2*, **n** *Cldn12*, **o** *Cldn16*, and **p** *Cldn19*, in the RNA-seq-based transcriptome analysis of ileum, kidney, and parotid glands (*n* = 4). Data is expressed as transcripts per kilobase million (tpm)

As expected, transcriptome data showed that the apical Ca^2+^ channel *Trpv5* (transient receptor potential cation channel vanilloid) is highly abundant in kidney but absent from ileum (Fig. 3a), since it is well known that extra-renal expression of the channel is limited to duodenum/jejunum and placenta [32]. *Trpv6* was also expressed in kidney (though at lower levels than *Trpv5*) but was absent in ileum (Fig. 3b), also in agreement with previous reports showing intestinal expression of *Trpv6* in mouse duodenum/jejunum and colon but not in ileum [58]. *Trpv6* (Fig. 3b) but not *Trpv5* (Fig. 3a) was found in parotid glands, fitting well with data obtained from human salivary glands [33].

The Ca^2+^-binding protein *Calb1*/calbindin-D28K was highly expressed in kidney whereas it was absent from ileum (Fig. 3c). *Calb2*/calbindin-D29K mRNA was not detected in any of the analysed tissues (data not shown), whereas *S100g*/*Calb3*/calbindin-D9K expression was about 2 orders of magnitude higher in kidney than ileum (Fig. 3d), in agreement with duodenum being the intestinal segment with highest expression of *Calb3* in humans [4]. None of the 3 *Calb* transcripts was detected in parotids, partially fitting a previous study reporting the absence of *Calb1* and *Calb3*, though *Calb2* expression, in human parotid glands [33]. Low mRNA levels of *Cabp1*/Ca^2+^-binding protein 1 were measured in all samples (Fig. 3e).

From the 3 Serca paralogues, the Ca^2+^ ATPases that transport Ca^2+^ from the cytoplasm into the sarco (SR)/endoplasmic reticulum (ER), *Atp2a2*/Serca2 and *Atp2a3*/Serca3 but not *Atp2a1*/Serca1 have been shown to be widely distributed [75]. Here, we found that *Atp2a1*/Serca1 was detected only in the transcriptome of parotid glands (Fig. 3f), whereas *Atp2a2*/Serca2 was more abundant in ileum and kidney than in parotid glands (Fig. 3g). Instead, *Atp2a3*/Serca3 expression was about 1 order of magnitude higher in ileum and parotid glands than in kidney (Fig. 3h). This pattern of expression fits partially with published data, as *Atp2a2* and *Atp2a3* (though not *Atp2a1*), were found in human parotid glands [33] and *Atp2a3* is known to be particularly abundant in intestine [75].

From the 4 paralogues of Pmca, the plasma membrane Ca^2+^ ATPase that mediates basolateral efflux of Ca^2+^, *Atp2b1*/Pmca1, and *Atp2b4*/Pmca4 are widely expressed whereas expression of *Atp2b2*/Pmca2 and *Atp2b3*/Pmca3 is restricted to few tissues [68]. *Atp2b1*/Pmca1 was the most abundant in the 3 analysed transcriptomes, and its expression was higher in ileum than in kidney and parotid glands (Fig. 3i). The levels of *Atp2b2*/Pmca2 (Fig. 3j) and *Atp2b4*/Pmca4 (Fig. 3k) were about 1 order of magnitude higher in kidney than in the other tissues, whereas *Atp2b3*/Pmca3 was not detected in any of the 3 organs (data not shown). Our data fits well with their general pattern of expression and with their expression in salivary glands of humans and rats [8, 33]. From the 3 Ncx2 paralogues, the basolateral Na^+^/Ca^2+^ exchanger, *Slc8a1*/Ncx1 is virtually ubiquitously expressed whereas *Slc8a2*/Ncx2 and *Slc8a3*/Ncx3 are restricted to lung and excitable tissues [42]. Accordingly, *Slc8a1*/Ncx1 mRNA was detected in all analysed tissues, though it was 2 orders of magnitude more abundant in the renal transcriptome than in ileum and parotid glands (Fig. 3i). The expression of *Slc8a2*/Ncx2 was below 0.2 tpm in all tissues, whereas *Slc8a3*/Ncx3 mRNA was not detected in any of the investigated samples, in agreement with their restricted tissue expression and their reported absence from human salivary glands [33].

From the claudins involved in paracellular transport of Ca^2+^, *Cldn2* (Fig. 3m) and *Cldn12* (Fig. 3n) were the ones with higher expression in ileum and kidney where their levels were 1 to 2 orders of magnitude higher than in parotid glands. This data is in agreement with the known role of both Cldns in intestinal and renal (re)absorption of Ca^2+^ [6]. Expression of *Clnd2* and *Cldn12* mRNA was recently reported in parotid glands of pigs [77], though a previous report failed to detect Cldn2 immunosignal in any of the salivary gland in rats [59]. Very low levels of *Cldn14* were observed in all analysed tissues (data not shown), consistent with the low expression of this negative regulator of paracellular Ca^2+^ permeability in the absence of hypercalcemia [16, 25]. In agreement with previous reports [39], mRNAs of *Cldn16* (Fig. 3o) and *Cldn19* (Fig. 3p), both responsible for familial hypomagnesemia with hypercalciuria and nephrocalcinosis [7, 73], were mostly restricted to kidney, though Cldn16 immunoreactivity has been reported at the basal membrane of acinar cells in human submandibular glands [45].

### Transcriptome analysis of genes involved in hormonal regulation of Pi and Ca^2+^ homeostasis in parotid glands, ileum, and kidney

**Fig. 4:**
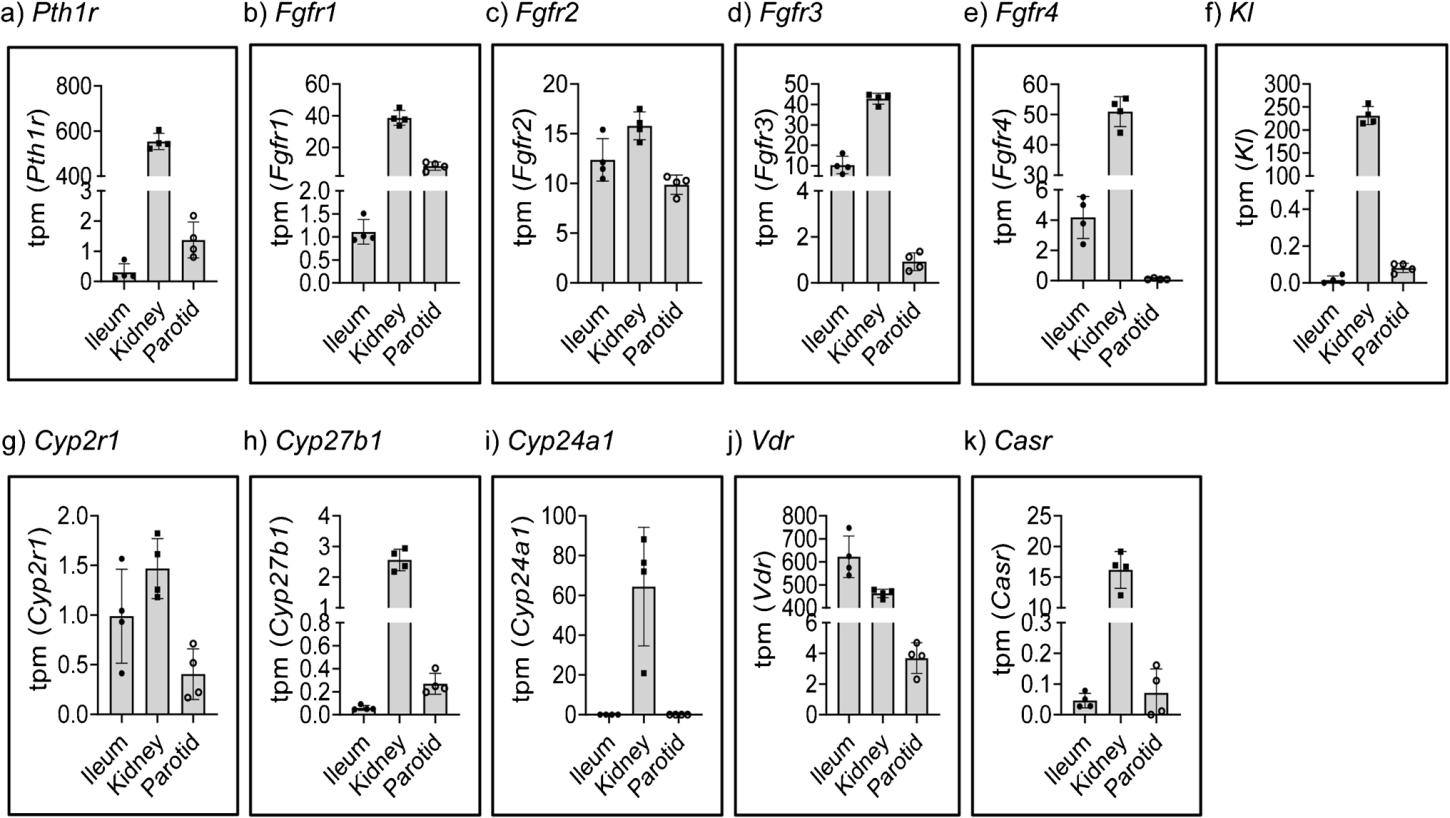
Transcriptome quantification of genes involved in regulation of Pi and Ca.^2+^ homeostasis in parotid glands, ileum, and kidney. Abundance of **a** the PTH receptor *Pthr1*, of Fgf receptors **b** *Fgfr1*, **c** *Fgfr2*, **d** *Fgfr3*, **e** *Fgfr4*, and **f** *Kl*, the klotho co-receptor, of vitamin D_3_ metabolising enzymes and receptors **g** *Cyp2r1*, **h** *Cyp27b1*, **i** *Cyp24a1*, **j** *Vdr*, and of **k** *Casr*, in the RNA-seq-based transcriptome analysis of ileum, kidney and parotid glands (*n* = 4). Data is expressed as transcripts per kilobase million (tpm)

Next, we compared the expression of the receptors of the main endocrine factors involved in hormonal regulation of Pi and Ca^2+^ transport, namely parathyroid hormone (PTH), fibroblast growth factor 23 (Fgf-23) and vitamin D_3_ (for review, see [36, 49, 52]). As expected, the mRNA expression of the PTH receptor *Pth1r* was more than 2 orders of magnitude higher in kidney than in ileum, with only low levels detected in parotid glands (Fig. 4a). *Pth2r* was hardly detected in any of the studied samples (data not shown).

Though extra-osseous expression of *Fgf-23* mRNA has been reported [14, 56], *Fgf-23* transcripts were not detected in any of the analysed tissues (data not shown). The kidney was the organ with the highest expression of FGF receptors (*Fgfr*s) mRNA, with similarly high levels of *Fgfr1* (Fig. 4b), *Fgfr3* (Fig. 4d) and *Fgfr4* (Fig. 4e), and slightly lower *Fgfr2* (Fig. 4c), a pattern that fits well with previously published data [2, 22]. *Fgfr2* and *Fgfr3* were the most abundant transcripts in ileum, whereas *Fgfr1* and *Fgfr2* were the mRNAs with higher expression in parotid glands (Fig. 4b-e). Protein expression of all 4 receptors has been reported in human ileum as well as along the whole intestine in mice, though Fgfr3 seems particularly enriched in crypts whereas Fgfr4 was detected in the embryonic gut [1] (for review, see [13]). The presence of *Pth1r* and *Fgfr1* and absence of *Fgfr3* and *Fgfr4* transcripts was recently reported in salivary glands of mice, but the expression of *Fgfr2* was not analysed [40]. As expected based on previous reports [46], while mRNA of the Fgf-23 coreceptor α*klotho (Kl)* was highly expressed in kidney, it was not detected in ileum (Fig. 4f); moreover, the coreceptor was barely expressed in parotid glands (Fig. 4f), in agreement with a recent report [40].

A number of hydroxylases are involved in the activation (*Cyp2r1*, *Cyp27b1*) and inactivation (*Cyp24a1*) of vitamin D_3_ (for review see [52]). As expected, all of them were highly or moderately expressed in kidney (Fig. 4g-i). Instead, they were either not detected or detected at very low levels in ileum and parotid glands, except for *Cyp2r1* transcripts the levels of which were comparable in all tissues. As expected, the vitamin D receptor *Vdr* mRNA was highly expressed in ileum and kidney, and it was also detected in parotid glands though at about 2 orders of magnitude lower levels than in the other organs (Fig. 4j). The expression of the Ca^2+^ sensing receptor (*Casr*) was more than 2 orders of magnitude higher in kidney than in the other tissues where it was hardly detectable (Fig. 4k) in agreement with a recent report [40].

### Short-term treatment with vitamin D_3_ does not alter the mRNA expression of Pi and Ca^2+^ transporters in salivary glands

**Fig. 5:**
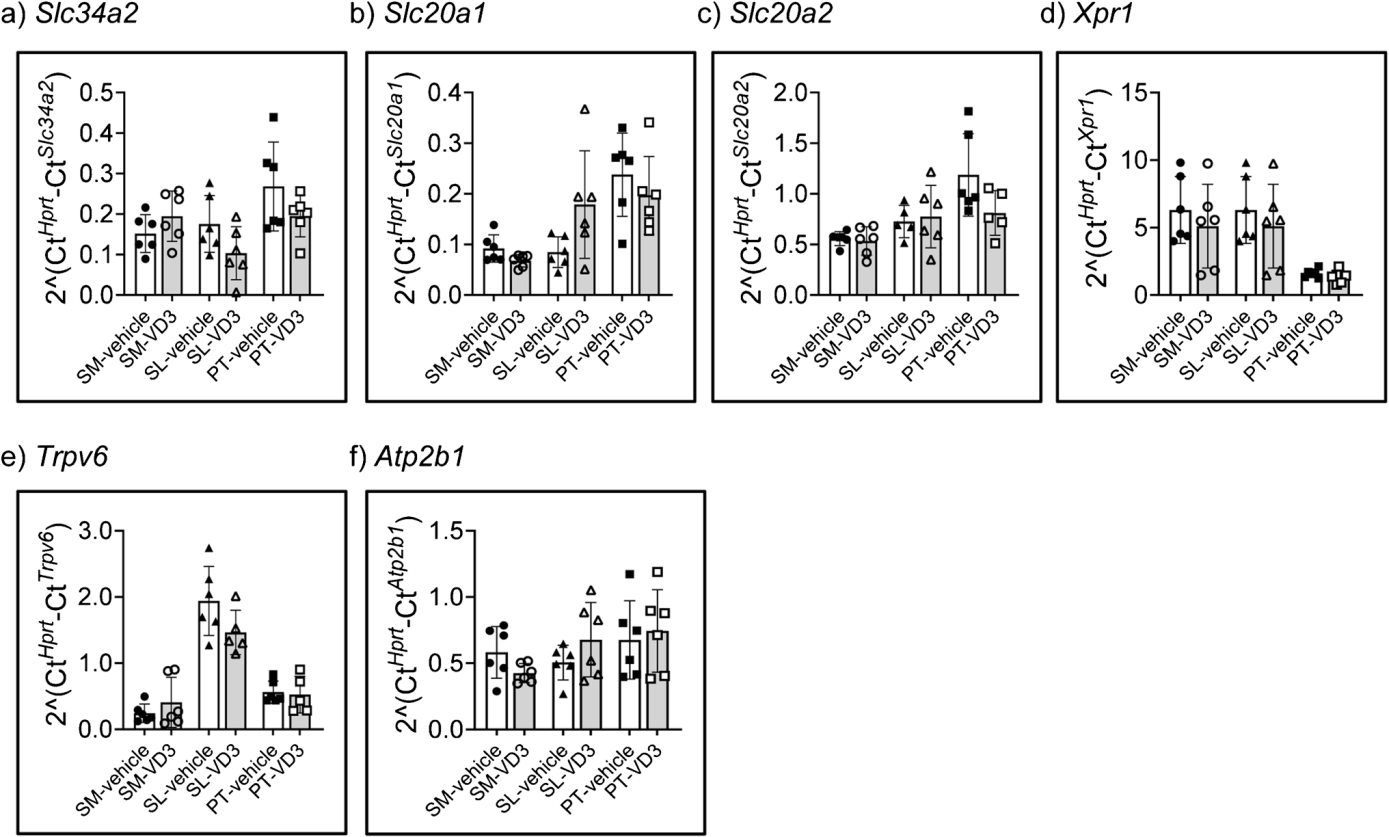
Effect of short-term vitamin D_3_ treatment on the expression of selected genes involved in regulation of Pi and Ca^2+^ homeostasis in submandibular, sublingual, and parotid glands. The expression of **a** *Slc34a2*, **b** *Slc20a1*, **c** *Slc20a2*, **d** *Xpr1*, **e** *Trpv6*, and **f** *Atp2b1* was quantified by qPCR in submandibular (SM), sublingual (SL), and parotid glands (PT) of mice injected once a day during two consecutive days with either vehicle or 4 μg kg^−1^ BW of 1,25(OH)_2_ vitamin D_3_ (VD3) (*n* = 6/group). Data was normalised to the expression of *Hprt*. Statistical analysis was done with Student’s *t* test; *P* ≤ 0.05 was considered as statistically significant

We next analysed the effect of vitamin D_3_ on the expression of selected genes in submandibular, sublingual and parotid glands. To confirm that the treatment had produced the expected systemic effect, the expression of the already mentioned hydroxylases involved in vitamin D_3_ metabolism was first analysed. As expected, the renal expression of the catabolic *Cyp24a1* was higher in mice injected with vitamin D_3_ as compared with vehicle-injected mice (1.45 ± 0.46 vs 0.21 ± 0.24, *t* test *p* = 0.0002; *n* = 6), whereas the expression of the anabolic *Cyp27b1* was lower in the vitamin D_3_-injected group (0.10 ± 0.07 vs 0.0022 ± 0.001, *t* test *p* = 0.005; *n* = 6), thus confirming the effectiveness of the treatment. Administration of vitamin D_3_ did not alter the mRNA expression of any of the apical Pi-transporters shown to be expressed in parotid glands (*Slc34a2*, *Slc20a1* and *Slc20a2*), neither that of the putative transporter mediating basolateral efflux (*Xpr1*) (Fig. 5a-d). Finally, vitamin D_3_ treatment did not alter either the mRNA levels of the apical (*Trpv6*) or basolateral (*Atpb1*/Pmca1) transporters of Ca^2+^ that were detected at higher levels in parotid glands transcriptome (Fig. 5e-f).

## Discussion

Transport of Pi across intestinal and renal epithelia has been analysed in detail, whereas handling by salivary glands is poorly understood. Intestinal Pi absorption depends upon passive/paracellular as well as active/transcellular processes, whereas Pi transport across the tight renal proximal epithelia relies only on the transcellular pathway ([5, 12, 43, 50], for review, see [29, 49]). In both tissues, active uptake of Pi across the BBM is mostly mediated by the Slc34 family of Na^+^/Pi cotransporters (*Slc34a2*/NaPi-IIb in intestine and *Slc34a1*/NaPi-IIa and *Slc34a3*/NaPi-IIc in kidney) with some potential minor contribution from Slc20 paralogues (for review, see [18, 49]). The identity of the molecule(s) responsible for paracellular transport in the intestine and for basolateral efflux in both epithelia remains to be fully elucidated, though Cldn3 [26] and Xpr1 [23] have been proposed to tighten the intestinal epithelia for Pi and to mediate basolateral efflux, respectively. The high concentration of Pi in the saliva suggests that salivary glands are able to concentrate Pi, and expression of NaPi-IIb has been reported in human parotid and submandibular glands, with the cotransporter localizing to either the basolateral (acinar cells) or the apical (ductal cells) membrane [34]. Though single-cell RNA-seq failed to detect *Slc34a2*/NaPi-IIb transcripts in mouse submandibular glands [67], immunofluorescence studies confirmed its apical expression in ductal submandibular cells of mice [40]. Here, we compared the transcriptome of parotid glands with those of ileum and kidney, to address their similarities/differences regarding the expression of genes involved in Pi homeostasis. Given the interconnections between Pi and Ca^2+^ metabolism, the expression of genes involved in Ca^2+^ homeostasis was also analysed.

Paracellular intestinal absorption of Pi has been reported along the whole intestine in mice [43], whereas active/transcellular absorption is restricted to particular segments in a species-specific manner, namely duodenum/jejunum in rabbits, rats, and humans and ileum in mice (for review, see [29]). Instead, renal reabsorption of Pi depends only on the transcellular route and transport takes place along the proximal tubule (for review, see [18, 49]). In addition to the well documented specific distribution of *Slc34* paralogues and ubiquitous expression of *Slc20* paralogues, the transcriptome analysis revealed high expression of *Cldn3* and *Xpr1* mRNA in ileum whereas *Xpr1* but not *Cldn3* was highly expressed in kidney. The low levels of *Cldn3* in kidney may indicate that this claudin is required to tighten a per se leaky epithelia such as the intestine. Unlike in intestine and kidney, *Slc20* paralogues seem the most abundant apical Pi transporters in parotid glands, though low mRNA expression of *Slc34a2*/NaPi-IIb was also observed and confirmed at the protein level. Moreover, parotid glands had a low expression of *Xpr1* and *Cldn3* mRNAs. Assuming that indeed Cldn3 tightens a leaky epithelium, its low levels in parotid glands may indicate that this tissue does not express the molecular components of the paracellular route. The absence of a paracellular Pi pore in salivary glands is physiologically coherent, since particularly in the more distal parts of the ducts such pore would result in passive transport of Pi back from the lumen (where its concentration may reach up to 13 mM in humans [60]) into the blood. On the other hand, the low expression of *Xpr1* in the salivary gland transcriptome may suggest the presence of additional transporters mediating basolateral efflux of Pi in ductal cells as well as apical efflux in acinar cells. Expression of Slc34a2/NaPi-IIb has been proposed to occur at basolateral sides in human acinar cells and at luminal sides in ductal cells [34]. We (this report) and others [40] confirm the luminal localization of NaPi-IIb in ductal cells but both failed to detect basolateral staining in murine glands. It remains to be clarified whether this represents a species difference or basolateral staining depends on the specificity of the antibody used. We verified the specificity of the antibody in ileum sections of intestinal-specific NaPi-IIb KO mice. Of note, in all healthy tissues examined to date, NaPi-IIb has been exclusively localized to apical domains but not to basolateral membranes [19, 70].

Transepithelial transport of Ca^2+^ in intestine and kidney involves active and passive components. The active transport depends upon the presence of apical channels that mediate luminal uptake (Trpv), intracellular Ca^2+^-binding proteins and pumps that prevent changes in cytoplasmic Ca^2+^ concentration (Calb, Cabp, and Serca), and basolateral pumps and exchangers that extrude Ca^2+^ across the basolateral membrane (Pmca and Ncx), whereas the passive route relies on claudins (for review, see [15, 31]). In the intestine, active Ca^2+^ transport predominates in duodenum and jejunum whereas passive absorption takes place along most of the intestinal tract. Accordingly, genes coding apical Ca^2+^ channels were not detected in the transcriptome analysis of ileum, whereas this tissue expressed high levels of *Cldn2* and *Cldn12*. Both Cldns are known to contribute to intestinal paracellular transport of Ca^2+^ and to be upregulated by vitamin D_3_ in vitro and in vivo [20]*.* The high expression of *Atp2a*/Serca (paralogues a2 and a3) and *Atp2b/*Pmca (paralogue b1) mRNAs detected in the transcriptome of ileum may be related to intracellular Ca^2+^ signalling rather than to transcellular transport. On the other hand, active transport of Ca^2+^ in the kidney occurs in the distal convoluted tubule and connecting duct whereas passive reabsorption takes place along the proximal tubule and thick ascending limb. Reflecting the presence of all nephron segments, the renal transcriptome analysis revealed the expression of all gene families involved in transcellular and paracellular transport of Ca^2+^, with particularly high expression of *Trpv5*, *Calb3*, *Atp2a2*, *Atp2b1*, *Slc8a1*, and *Cldn2*/*Cln12*. Similarly, apical channels (*Trpv6*) as well as Cldn (*Cldn2* and *Cldn12*) were detected in the transcriptome of parotid glands, suggesting the presence of both transport pathways in this salivary tissue. Interestingly, intracellular Ca^2+^ buffering in parotid cells seems to depend not on the Calb family but on the Cabp1, whereas transport into ER does not seem to rely only on the ubiquitous Serca paralogues (*Atp2a2* and *Atp2a3*) but also on the Serca1/*Atp2a1*, a pump whose expression was suggested to be restricted to fast-twitch cardiac striated muscles [75].

**Fig. 6:**
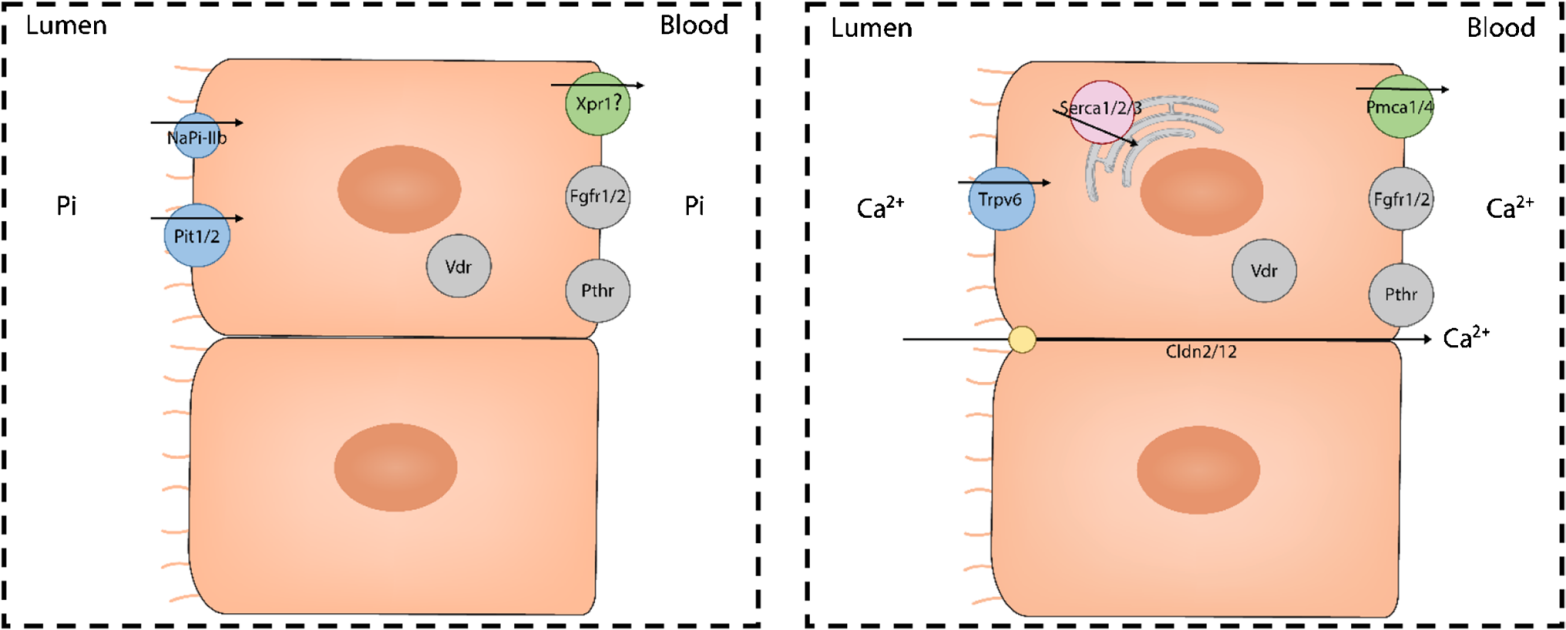
Hypothetical model of a parotid epithelial cell showing the expression of transporters and regulators involved in Pi (left) and Ca^2+^ (right) homeostasis based on the transcriptomic data. ©gritsalak/Adobe Stock, done in Adobe Illustrator

To study whether parotid glands can be a target for hormones involved in Pi (and Ca^2+^) metabolism, we analysed the expression of receptors for their main endocrine regulators, namely PTH, FGF-23 and vitamin D_3_ (for review, see [36, 49, 52]. PTH regulates plasma levels of Pi and Ca^2+^ by acting on bones, intestine and kidneys. Its effects are mediated by the Pthr1 receptor, a member of the G protein-coupled receptor superfamily that also binds PTH-related protein (PTHrP); a second receptor (Pthr2) was identify by homology-cloning, but later experiments showed that it had a very low affinity for PTH or PTHrP (for review, see [21]). Although at low level compared with kidney, parotid glands also expressed mRNA for the PTH receptor *Pth1r*. Indeed, PTH has been reported to have a direct effect in parotid glands by increasing the concentration of Pi in parotid saliva in vascularly isolated parotid glands of sheep as well as in parotid cell aggregates of rats [65, 74], thus indirectly implying the presence of the hormonal receptor in the tissue. Instead, *Pth1r* mRNA was hardly detected in ileum, suggesting this segment is not a direct target of PTH, though the PTH status indirectly influences intestinal transport of Pi and Ca^2+^ by regulating the circulating levels of vitamin D_3_. Fgf-23 also controls Pi and Ca^2+^ homeostasis by modulating their renal reabsorption. Its effects depend upon binding to receptors of the tyrosine kinase family, and in most cases require the presence of αklotho as a co-receptor (for review, see [36]). Parotid glands expressed moderate levels of *Fgfr1* and *Fgfr2* transcripts; however, the near absence of αklotho mRNA makes it unlikely for these salivary glands to be responsive to FGF-23 unless circulating soluble αklotho is available to bind to FGF receptors. A similar argument can be made for ileum since, despite moderate gene expression of some Fgfr, klotho was absent. Vitamin D_3_ regulates renal and/or intestinal transport of Pi and Ca^2+^ by stimulating the expression of particular transporters [20, 27, 32]. The transcriptional effects of vitamin D_3_ are mediated by the Vdr, a receptor from the nuclear receptor family of transcription factors. The transcriptome data revealed low but consistent mRNA expression of the *Vdr* in parotid glands. Figure 6 shows a hypothetical model of parotid cells showing the expression of transporters and regulators involved in Pi (left) and Ca^2^^+^ (right) homeostasis based on the transcriptomic data.

Although the transcriptome analysis suggested that parotid glands can be a target for vitamin D_3_, we found that short-term administration of vitamin D_3_ to mice had no effect on the gene expression of the analysed Pi and Ca^2+^ transporters. In this regard, vitamin D_3_-induced upregulation of intestinal NaPi-IIb is proposed to associate with transcriptional changes in young but not adult rodents [76], and Vdr-deficient mice have low levels of NaPi-IIb protein without changes at the mRNA level [63]. The responsiveness of Slc20 paralogues to vitamin D_3_ administration is probably tissue-specific, whereas a single report suggests that Xpr1 is not responsive [28, 41, 69]. On the other hand, the mRNA expression of intestinal *Trpv5* but not of *Pmca1* is reduced in Vdr-deficient mice [71], with both transcriptional stimulation [3] and unchanged mRNA levels of *Pmca1* [28] reported in vitamin D_3_ treated mice. Nevertheless, it should be noticed that the vitamin D_3_ status was previously shown to affect parotid gland function in rats, with the rate of parotid salivation as well as the content of Ca^2+^ and Pi in parotid saliva reduced in rats with chronic vitamin D deficiency [24].

Current models of saliva production state that formation of primary saliva is driven by ion secretory processes in acinar cells leading to the secondary water movement into the luminal space. Once primary saliva is formed, reabsorption of Na^+^ and Cl^−^ and secretion of HCO3^−^ and K^+^ in the absence of water permeability along the salivary ducts forms hypotonic, bicarbonate-rich secondary saliva. This saliva contains higher concentrations of Pi than plasma. At this stage, it remains unclear how Pi is first translocated across the epithelium into the primary saliva and then enriched in the final saliva. The latter is likely not due to the reabsorption of water as the ductal epithelium is considered to be highly water-impermeable. Our data confirm and expand previous observations that salivary glands express a series of proteins that are involved in other epithelia in Pi (re)absorption. However, our findings provide no explanation for the high concentration of Pi in final saliva as all transport proteins described here would favour reabsorption due to electrochemical gradients. This would suggest that unknown mechanisms must exist that mediate the secretion of Pi into saliva against its chemical gradient.

In summary, our data suggest that (a) parotid glands are equipped preferentially with *Slc20* rather than with *Slc34* Na^+^/Pi cotransporters and express very low levels of *Cldn3* and *Xpr1* mRNAs, the current candidates for regulation of paracellular permeability and basolateral transport of Pi; (b) parotid glands are susceptible to transport Ca^2+^ through the transcellular and paracellular route; (c) parotid glands are potential targets for PTH and vitamin D_3_ regulation.

## Supplementary Information

Below is the link to the electronic supplementary material.

Supplementary file1 (PDF 124 KB)

Supplementary file2 (XLSX 7.67 MB)

## Data Availability

All supporting data are included in the manuscript and supplements.
