# Supplementary material for: Expression of phosphate and calcium transporters and their regulators in parotid glands of mice
Source: Pflugers Arch. 2022 Oct 24;475(2):203–16. doi: 10.1007/s00424-022-02764-x (PMC9849193; doi:10.1007/s00424-022-02764-x)

Table 1

Gene	Fw primer	Rv primer	Probe	Reporter	Quencher	Amplicon size (bp)
Slc34a1/NaPi-IIa	TGATCACCAGCATTGCCG	GTGTTTGCAAGGCTGCCG	CCAGACACAACAGAGGCTTCCACTTCTATGTC	FAM	TAMRA	133
Slc34a2/NaPi-IIb	CTTGGGACCTGCCTGAAC	AATGCAGAGCGTCTTCCCTTT	TGGTCAGAGAGAGACAC	FAM	TAMRA	77
Slc34a3/NaPi-IIc	CAGCGGTATTACCAGCAACA	CTGTCCTCCTCTGGAGATGC	GTGGCCTCTTCAGCTCTTGCACAGA	FAM	TAMRA	150
Slc20a1/Pit1	CGCTGCTTTCTGGTATTATGTCTG	AGAGGTTGATTCCGATTGTGCA	TTGTTCGTGCGTTCATCCTCCGTAAGG	FAM	TAMRA	135
Slc20a2/Pit2	AGGAGTGCAGTGGATGGAGC	ATTAGTATGAACAGCACGCCGG	ATTGTCGCCTCCTGGTTTATATCGCCAC	FAM	TAMRA	96
Xpr1	CCAGAAGAACCGGTCGTG	CGTTGGTCGAGGAAAGGA	CCTGGCTTCCCAATCCAAGGCTC	FAM	TAMRA	190
Slc17a4						
Cyp24a1	CAAATCAGTCAAACCCTGCAT	GGCGTACAGTTCCTTCTTGG	TACAGAGATATTCCCAGCAGCCCG	FAM	TAMRA	190
Cyp27b1	Premixed Mm01165916 _ g1 (Thermo Fisher Scientific)					
Vdr	AGGCCCACACTCAGCTTCT	ACAGGTCCAGGGTCACAGAG	TACACCCCCTCACTGGACATGATGG	FAM	TAMRA	145
Trpv6	CTGGAGAGCACAGGTTGTGG	CCAAGACCATACTCTCGCCC	-	-	-	102
Trpc3	TGGGATACTCAAAGTCCAGGTTAA	TGAGAATGCTGTTAAAACTGTGTGA	-	-	-	
Trpc6	CTACATTGGCGCAAAACAGA	AGACCAAAGATAGCCCAGAACA	TGAAGCATTCACAACAGTTGAGGAAAGTTTT	FAM	TAMRA	81
Atp2b1	AAATTCCTTCAGTTCCAGCTT	GTAGCCAGAGCCAGGGAAG	CAAGACTCGCCACTTAAGGC	FAM	TAMRA	152
Hprt	TTATCAGACTGAAGAGCTACTGTAATGATC	TTACCAGTGTCAATTATATCTTCAACAATC	TGAGAGATCATCTCCACCAATAACTTTTATGTC	VIC/FAM	BHQ/TAMRA	127

Supplementary Figure 1

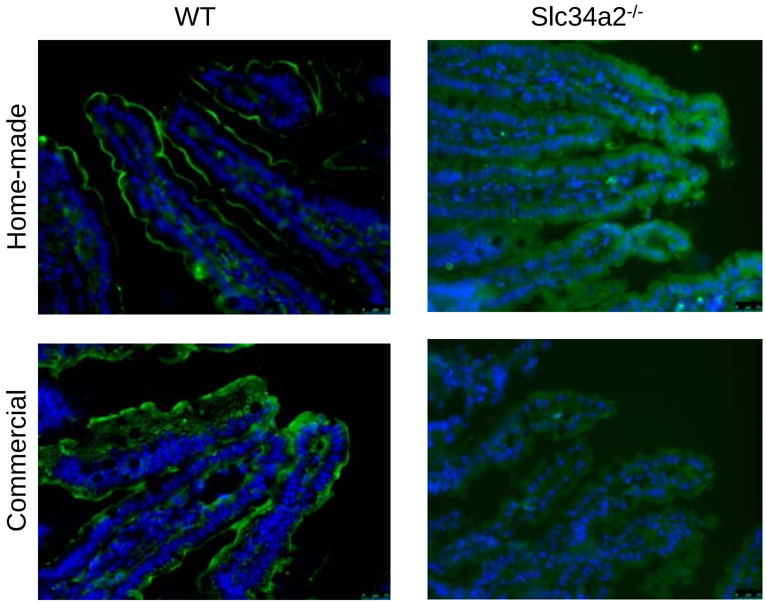

Supplement: Supplementary file 1 — Supplementary file1 (PDF 124 KB) [file 424_2022_2764_MOESM1_ESM.pdf]
